# Xenogeneic Heterotopic Auxiliary Liver transplantation (XHALT) promotes native liver regeneration in a Post-Hepatectomy Liver failure model

**DOI:** 10.1371/journal.pone.0207272

**Published:** 2018-11-21

**Authors:** Nalu Navarro-Alvarez, Zurab Machaidze, Christian Schuetz, Alexander Zhu, Wei-hui Liu, Jigesh A. Shah, Parsia A. Vagefi, Nahel Elias, Leo Buhler, David H. Sachs, James F. Markmann, Heidi Yeh

**Affiliations:** 1 Center for Transplantation Sciences, Massachusetts General Hospital/Harvard Medical School, Boston, MA, United States of America; 2 University of Geneva School of Medicine, Geneva, Switzerland; Indiana University, UNITED STATES

## Abstract

The liver’s regenerative capacity is unique, but too small a segment can overwhelm its ability to simultaneously regenerate and support the host, resulting in liver dysfunction and death. Here we tested a temporary Xenogeneic Heterotopic Auxiliary Liver Transplant (XHALT) from Gal-KO miniature swine in a baboon model of Post-Hepatectomy Liver Failure (PHLF) by 90%- hepatectomy. Immunosuppression consisted of CVF, ATG, FK 506 and steroids. 90%-hepatectomized animals died within 4–5 days with the clinical picture of PHLF, (high LFTs and bilirubin, ascites, encephalopathy and coagulopathy). The 10% remnants had macroscopic and histological evidence of severe steatosis and absence of hepatocyte replication. In contrast, the addition of XHALT prolonged survival up to 11 days, with the cause of death being sepsis, rather than liver failure. The remnant liver appeared grossly normal, and on histology, there was no evidence of fatty infiltration, but there was pronounced Ki-67 staining. In conclusion, temporary auxiliary xenografts have the potential to support a small for size liver graft while it grows to adequate size or provide an opportunity for organ recovery in acute liver failure.

## Introduction

Donor organ shortage remains a barrier to organ transplantation. As of 2016, it is estimated that on average, 21 people die each day while waiting for a transplant in the United States. There are currently 14,789 patients on the waiting list for a human donor liver; of these, only 7,841 received a transplant (http://www.unos.org) (last accessed on 1.19.2017).

While living donor liver transplantation (LDLT) has somewhat extended the liver donor pool, there is the associated risk of "Small-For-Size Syndrome" (SFSS) or (PHLF), where the liver cannot maintain vital functions due to insufficient cell mass. The liver loses its ability to regenerate, resulting in delayed synthetic dysfunction, prolonged cholestasis, coagulopathy, ascites, and high mortality [1]. In order to avoid SFSS in the recipient, 40% of the standard liver volume [2] or 0.6–0.8% of the graft weight to patient weight ratio [3] is generally considered to be the minimal graft volume required. However, grafts of this size can put the donor at risk of liver failure [4,5]. Thus, LDLT is a limited solution for the shortage of deceased donor livers [6,7].

Xenotransplantation is an attractive strategy to compensate for this donor shortage [8]. Although there are still several issues that need to be addressed for this therapy to become a reality, α-1,3-galactosyltransferase gene-knockout (GT-KO) pigs that do not express the α-1,3Gal (Gal) antigens against which most primate anti-pig antibodies are directed, have improved the likelihood of this potential therapy [9]. Our recent reports demonstrating the survival of a GT-KO pig liver xenograft for more than 25 days, supports the notion that pig livers could be used as bridge therapy while the native liver regenerates, or as a temporary support for small segments [10,11].

We therefore tested the effect of a Xenogeneic Heterotopic Auxiliary Liver Transplantation (XHALT) from Gal-KO pigs into baboons undergoing 90% hepatectomy and report our observations in this pilot study.

## Materials and methods

### Ethics statement

The animal procedures and care in this study was in accordance with the NIH guidelines for the Care and Use of Laboratory Animals and approved by the Massachusetts General Hospital (MGH) Institutional Animal Care and Use Committee (protocol approval number 2010N000177). All surgery was performed under ketamine and Isofluorane anesthesia, and all efforts were made to minimize suffering including the use of pain medication which was performed with fentanyl and buprenorphine.

### Experimental animals and general welfare of the non-human primates

Baboons (Papio hamadryas n = 5, Mannheimer Foundation, FL) 8–10 kg were used as recipients, and Gal-KO miniature swine 5-7kg as donors.

The animals were housed in the facilities of the Center for Transplantation Sciences which is fully accredited by the Association for Assessment and Accreditation of Laboratory Animal Care International. Animals were on automatic 12hr light/dark cycles (7am/7pm) and they were monitored daily for health, humane treatment and husbandry conditions. Baboons were given biscuits in the mornings and evenings, and additionally they were offered fresh fruits and vegetables. Animals had unlimited access to water through a lixit. Baboons were provided with enrichment such as toys inside and hanging on their cages. In addition, they had auditory stimulus with TV and music playing. Animals were closely monitored for signs of clinical and general distress and a humane endpoint criterion was used to determine study endpoint which was done with injection of sodium pentobarbital 100mg/kg IV.

### Humane endpoints

The animals undergoing liver failure usually present with low appetite, the supportive care provided to these animals is the administration of ensure and yogurt with a syringe directly in their mouth. If we determined that the animals have nothing PO for 24hrs starting on D1 after surgery and no solid food for 72 hrs starting as well on D1 after surgery, then these were considered euthanasia endpoints.

In more detail, these were the criteria that we used to consider euthanasia immediately:

1. If the animal presented severe alteration of mental status that was refractory to urgent care treatment: (i.e animal down in the bottom of the cage, non-responsive to external stimuli, vocalization). Urgent care treatment could include (diuretics, electrolyte replacement, glucose supplementation, dopamine, IV fluids etc.)
2. If the animal presented severe ascites that was affecting the respiratory rate, it is refractory to urgent care treatment and needs to be tap for more than 2 times in a 24hr period, however the number of ascites taps allowed is a decision that was made in consultation with the veterinarian.
3. If the respiratory rate was less than 8 breaths/min or greater than 100 breaths/min that was refractory to urgent care treatment and if it did not respond to paracentesis, but this again was a decision made in consultation with the veterinarian.
4. If the animal required more than 200cc/blood/day (6U for 2 consecutive days) starting on POD1, this however was a decision that was made in consultation with the veterinarian.

### Monitoring and alleviation of pain and distress

After any surgical procedure: a member of the staff designated in the procedure form usually remains with the animal until fully recovered from the anesthetic, meaning until the animal is awake and moving purposefully around its cage. The animals are carefully monitored for any signs of pain or distress. Additional analgesics, other medications, and IV fluids will be administered as needed. In the immediate post-operative period, the animals are monitored 2–3 times a day, for the first 24-48hrs. However, if the clinical condition of the animal required more than 48hrs of often monitoring, this was provided as long as was needed. In addition, a veterinarian was on call 24 hours for consultation. The animal could be continuously monitored via a closed-circuit TV system. To alleviate any signs of pain or distress, animals were placed on a fentanyl patch which lasted for 3 days and buprenorphine 0.01 mg/kg IV or IM, single dose.

### Experimental design

Solid food was withheld for 12 hours before any surgical procedure, though the animal had unlimited access to water throughout this period. All surgical procedures were done under general anesthesia with Ketamine 20mg/kg IM or 10mg/kg IV single dose and Isofluorane 0.5–1.5% given continuously during the surgery. At the beginning of the procedure, 0.005 to 0.01 mg/kg of Buprenorphine was injected intramuscularly, followed by 0.005 to 0.01 mg/kg IM and the administration of a fentanyl patch (1-4ug/kg/hr) which duration of analgesia lasts for 72hrs. Additional analgesia was considered if the animal was observed to be in distress, with decreased appetite, and increase respiratory rate. Animals were divided into a control group (n = 3) undergoing 90%Hx, and an experimental group (n = 2) undergoing 90% Hx plus XHALT. Immunosuppression for animals in group 2 consisted of tacrolimus (FK) (0.2mg/kg) on D-2, ATG (10mg/kg) and Cobra Venom Factor (CVF) (100u/kg) on D-1 and FK continuous IV for a target serum level of 10-20ng/ml and methylprednisolone in a tapered fashion started at (120mg/d IV). For antimicrobial prophylaxis, cefazolin was administered after line placement, and then ganciclovir and ampicillin-sulbactam started at the time of transplantation. IV omeprazole was given for ulcer prophylaxis. Animals underwent intensive clinical observation throughout the course of the study and IV fluids were administered as required based on clinical and biochemical parameters.

### Donor liver procurement

Livers were obtained from MGH GalT-KO miniature swine donors that were derived through breeding of previously cloned founders [9]. On the day of transplant, liver donor procurement was performed as previously described [12]. In brief, after exposing the liver, it was flushed in situ through the aorta with 1L of cold Lactated Ringer’s followed by 1L of cold University of Wisconsin (UW) solution. The liver was then excised with the celiac trunk and infrarenal aorta in continuity, the portal vein (PV) to the confluence of the splenic (SV) and superior mesenteric veins (SMV), cuffs of supra- and infrahepatic cava, and the extra-pancreatic bile duct. Portal flush with UW, donor cholecystectomy, and preparation of the vessels were performed on the back table. The liver was stored on ice until implantation. This was a terminal procedure, so animal was euthanized following liver procurement.

### 90% hepatectomy and xenogeneic heterotopic auxiliary liver transplantation

All Baboons were jacket and tether trained (Lomir, Malone, NY, USA) previous to any experimental procedure. Baboon recipients underwent 90% Hx as a model of PHLF; the median, right and left lateral lobes comprising approximately 50%, 30% and 10% respectively were removed, leaving only the caudate lobe comprising the remaining 10% [13]. Following 90% Hx, animals in the experimental group underwent XHALT. First, splenectomy was performed. The donor liver was then implanted in the left upper quadrant by anastomosing the donor PV to the confluence of the recipient inferior mesenteric vein (IMV) and SV and the donor infrahepatic cava to the recipient left renal vein (LRV). On reperfusion, 100 cc of blood was flushed through the suprahepatic cava prior to ligating it. The donor infrarenal aorta was then anastomosed to the recipient infrarenal aorta for arterial perfusion of the auxiliary liver. External biliary drainage was with a T-tube into the donor bile duct as previously described [14]. A central line was used in both groups to access for fluids, medication administration and blood draws.

### Postoperative monitoring

Daily blood sampling was performed to asses for complete blood count (Hemavet 950 FS Drew Scientific Group; Waterbury, CT, USA), and chemistry for estimation of liver enzymes and electrolytes (Catalyst Dx; IDEXX, Holliston, MA, USA). FK trough levels were drawn daily prior to the administration of the morning dose. Clotting studies were performed in the clinical special coagulation laboratory at MGH.

### Monitoring of graft rejection and efficacy of immunosuppression

Blood samples were taken from animals undergoing XHALT before and after implantation, in order to monitor effectiveness of immunosuppression and potentially prevent graft rejection. Analysis was performed using flow cytometry. The following antibodies were used to stain peripheral blood: anti-CD3 (clone SP34-2), (BD Pharmingen) anti-CD20 (clone L27), (BD Biosciences). All samples were collected on a FACSCalibur (becton Dickinson, Mountain View, CA) and analysis performed with the FlowJo (TreeStart, Ashland, OR).

### Tissue sample collection and histological evaluation

Liver biopsies from the native liver were obtained under direct vision immediately after 90% Hx and from the transplanted liver on the back table and 30 and 60 minutes post-reperfusion. Additional biopsies of both livers were taken at the time of exploratory laparotomy performed for indication and at necropsy at the time of euthanasia.

Liver tissues were stored in 10% formalin, paraffin embedded, and stained with hematoxylin and eosin (H&E) and for Ki67 (DAKO M7240) to assess for liver regeneration. Images were viewed at 40X magnification and captured using a Nikon confocal microscope (Nikon D-ECLIPSE C1, Tokyo, Japan).

### Statistical analysis

Data are presented as mean ± SEM. Significance of the difference between two groups was determined by paired Student’s t test. Statistical analysis was performed with Prism software. Values of p<0.05 were considered significant.

## Results

### Clinical course after 90% Hx alone

**Fig 1:**
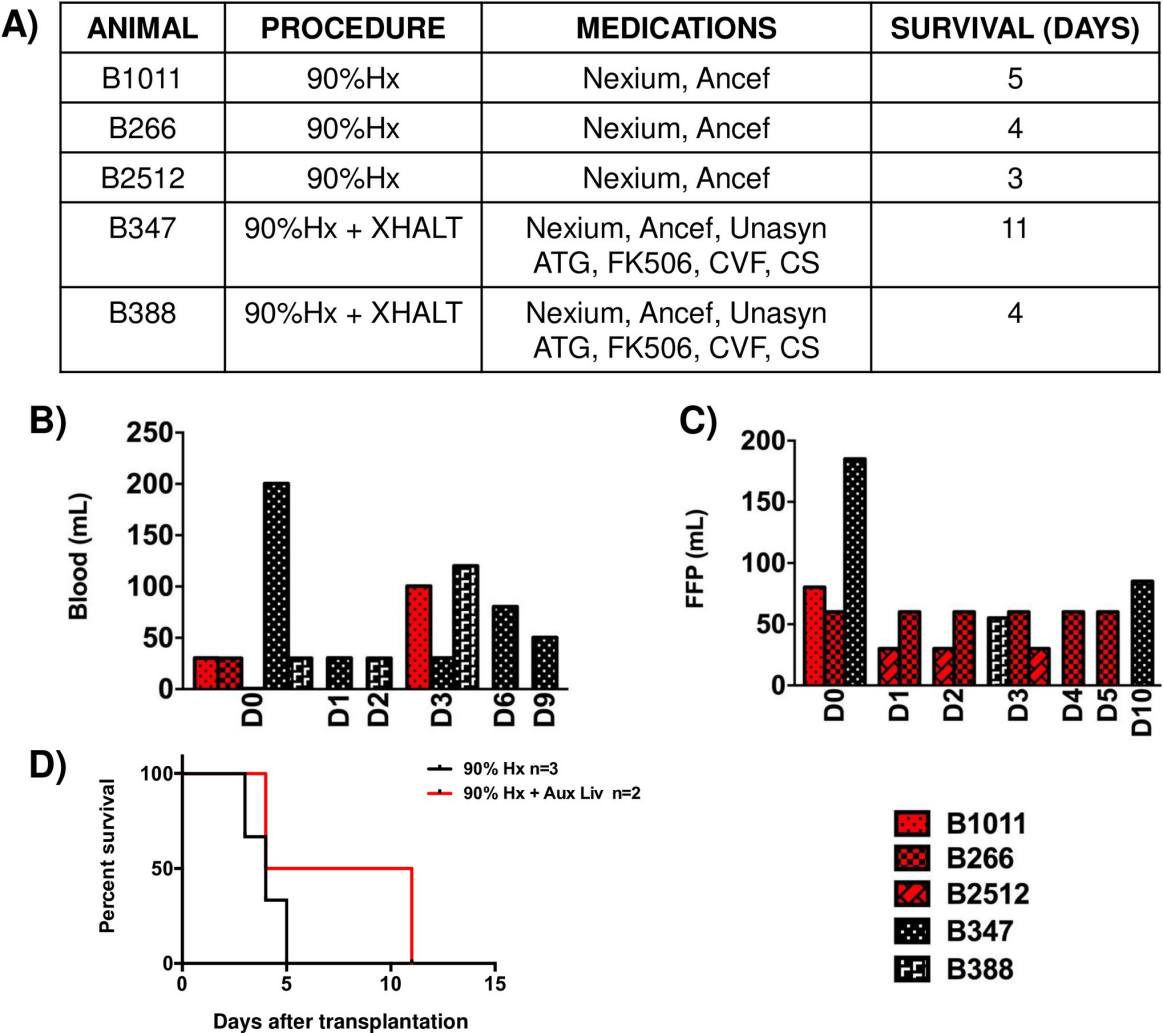
Auxiliary liver transplantation in 90% hepatectomized baboons. (A) Table depicting the procedures performed to each animal, the medications administered and the survival length. (B) Blood and (C) Fresh Frozen Plasma requirements throughout the survival of the animals. (red bars indicate animals undergoing 90% Hepatectomy alone; black bars, 90%Hx plus the addition of an auxiliary pig liver xenotransplantation (XHALT). (D) Survival of the animals.

**B1011** recovered slowly from surgery, with decreased appetite and activity. On POD3, the animal was re-explored due to bleeding from the midline incision. An organized blood clot was found on the cut surface of the liver, but no active bleeding. The liver was yellow. The abdomen was washed out and closed but unfortunately the animal could not be weaned off the ventilator and had to be euthanized on POD4 (Fig 1A and 1D). The animal required a total of 130ml of pRBCs over those 4 days (Fig 1B).

**B266** recovered fully after surgery and was quickly perched. However, as the day progressed, the animal's activity did not increase, she had no appetite and had low urine output. On POD2 the animal developed some ascites, which significantly increased until POD 5, when severe respiratory distress caused by the abdominal distension appeared. After therapeutic paracentesis with light sedation, the animal passed away later that day without recovering consciousness following the procedure (Fig 1A and 1D). This animal received a total of 30ml of pRBCs (Fig 1B).

**B2512** woke slowly and ate very little, having severe emesis on POD2. The animal deteriorated rapidly and was euthanized on POD3 for profound encephalopathy (Fig 1A and 1D). This animal did not required pRBC (Fig 1B).

### Clinical course after 90% hepatectomy plus XHALT

**Fig 2:**
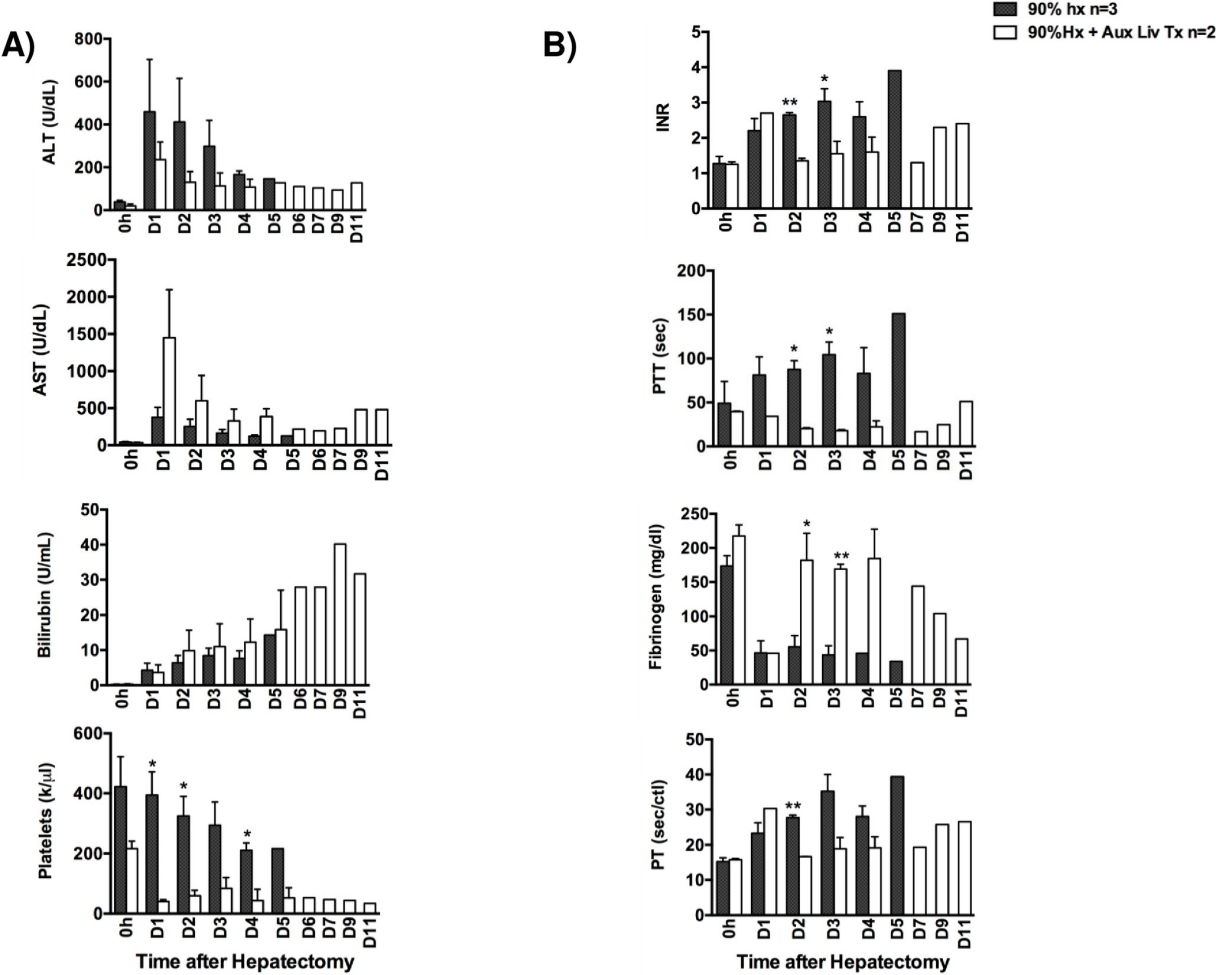
Liver function test. (A) Animal’s liver function tests were evaluated in peripheral blood: ALT, AST, Bilirubin as well as platelets until the animal’s demise. (B) blood coagulations tests were also evaluated by means of INR, PTT, Fibrinogen, and PT. Black bars indicate 90% Hepatectomy alone group. White bars indicate 90% hepatectomy plus Xenogeneic Heterotopic Auxiliary Liver Transplantation (XHALT).

**Fig 3:**
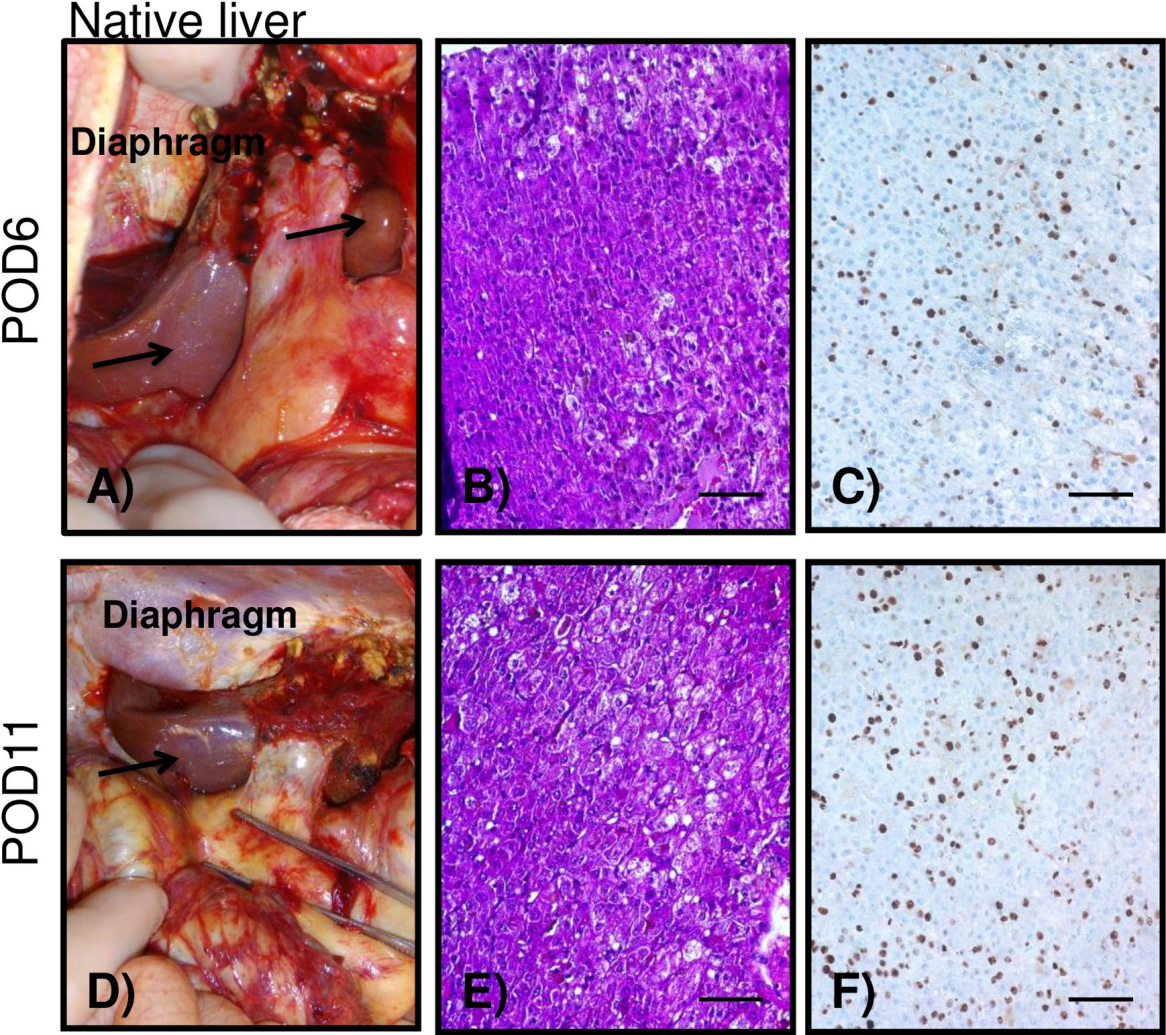
Native liver evaluation. Macroscopic and histological appearance of the native liver from animal B347 undergoing 90%Hx+ XHALT at the time of exploratory laparotomy (A-C) and on autopsy (D-F). Both time points demonstrating normal healthy-looking livers and with a large number of Ki67+ cells.

**Fig 4:**
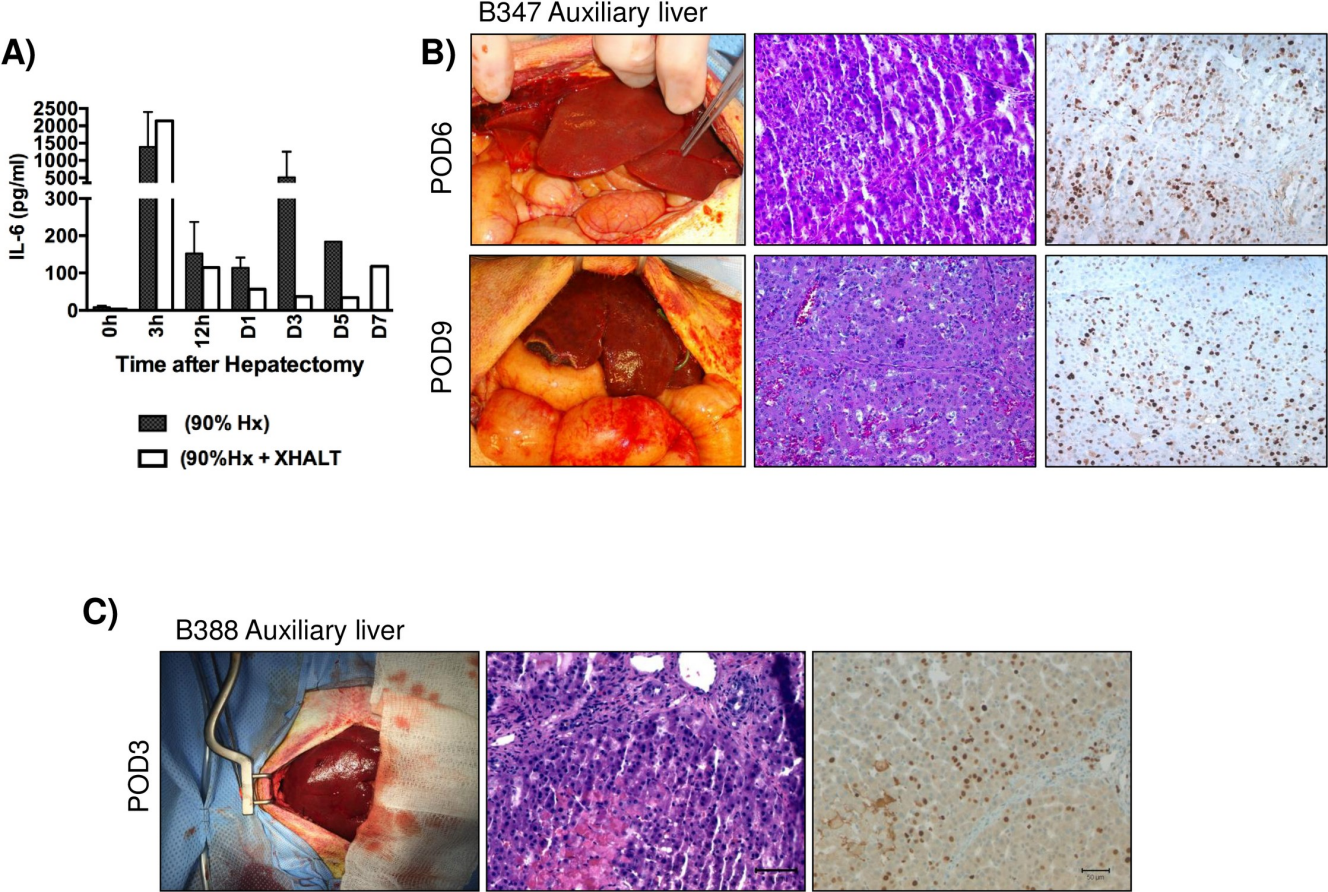
Xenogeneic auxiliary liver evaluation. (A) Serum IL-6 levels in both groups. (B,C) Macroscopic and histological appearance of the xenogeneic auxiliary liver from animals undergoing 90%Hx+ XHALT at the time of exploratory laparotomy and at graftectomy. Both animals at laparotomy demonstrated a large number of Ki67+ cells (B-C).

**B347** recovered from surgery slowly, had poor appetite and activity through POD2, but slowly became more active and hungry on POD3. A rise in bilirubin prompted an exploratory laparotomy on POD6 (Fig 2A). No bile leaks/obstructions were found, and both the graft and native liver looked normal and viable (Figs 3A and 4B). The animal returned to the OR on POD9 for another rise in Tbi and AST and lack of bile output in the XHALT drain (Fig 2A). 400ml of bloody ascites and 35 ml of bloody pleural fluid were drained. The native liver appeared normal, and slightly larger in size, however the donor liver xenograft was dark and firm, leading to graftectomy (Fig 4B). The left renal vein was damaged during graft removal, requiring kidney removal. The animal recovered poorly after anesthesia and developed severe hypoglycemia. By POD11, the animal was anuric and lethargic, and was euthanized (Fig 1A and 1D). On necropsy, the native liver appeared normal and had grown significantly in size (Fig 3). Ascites cultures from POD 9 eventually grew out pseudomonas. Due to low hematocrit and platelets, the animal required blood support throughout (Fig 1B).

**B388** The animal recovered well after surgery and had good appetite and activity for 3 days, in spite of getting daily general anesthesia for blood work, as his blood draw lines were not functioning. On POD3, the animal returned to the OR for a combination of mild abdominal distention and tachypnea, decreased bile output, and for placement of new lines. On exploration, there was blood tinged ascites and the xeno- and native livers were both healthy appearing. The bile tube was taken out of the bile duct, flushed, and replaced, and bile was observed to fill the tube. The animal recovered, but remained quiet throughout the day and was finally euthanized for abdominal distension and respiratory distress (Fig 1A and 1D). Autopsy showed bloody ascites, normal appearance of both native liver and the xenograft, and no evidence of vascular thrombosis (Fig 4C). Severe pulmonary edema was observed in both lungs and thought to be the cause of the animal’s demise. The animal required 180ml of pRBCs (Fig 1B).

### Improvement of liver injury markers with XHALT

Control animals showed continued increase in AST and ALT (Fig 2A) until death POD3-5. In contrast, animals receiving XHALT in addition to 90% Hx, had consistently lower ALT (Fig 2A) than control animals, in spite of a much larger hepatocyte mass in XHALT animals. AST, which is preferentially released by pig livers, were much higher in XHALT animals because of the presence of the xenograft, but decreased over time as the xenograft recovered, rather than continuing to increase as both the ALT and AST did in control animals (Fig 2A).

### Improvement of liver synthetic function parameters with XHALT

To determine the synthetic ability of the liver, coagulation parameters such as PT, PTT, INR and fibrinogen were measured. Control animals had a significant increase in INR, PT and PTT and associated decrease in fibrinogen starting on POD1 (Fig 2B), in spite of transfusing several units of FFP (Fig 1C).

XHALT animals also had increased INR, PT, and PTT on POD1, but this immediately decreased back to baseline by POD2 (Fig 2B). In addition, both XHALT recipients had normal fibrinogen levels by POD2, reflecting intact synthetic function of the combined livers, (Fig 2B) while receiving less FFP than control animals (Fig 1C). Of note, in spite of improved coagulation factor levels in the XHALT animals, they still required pRBC transfusions (Fig 1B), possibly related to severe thrombocytopenia (Fig 2A), which is a well-described finding in non-human primates receiving pig livers [15] [14].

### Decreased steatosis in liver remnant with XHALT

**Fig 5:**
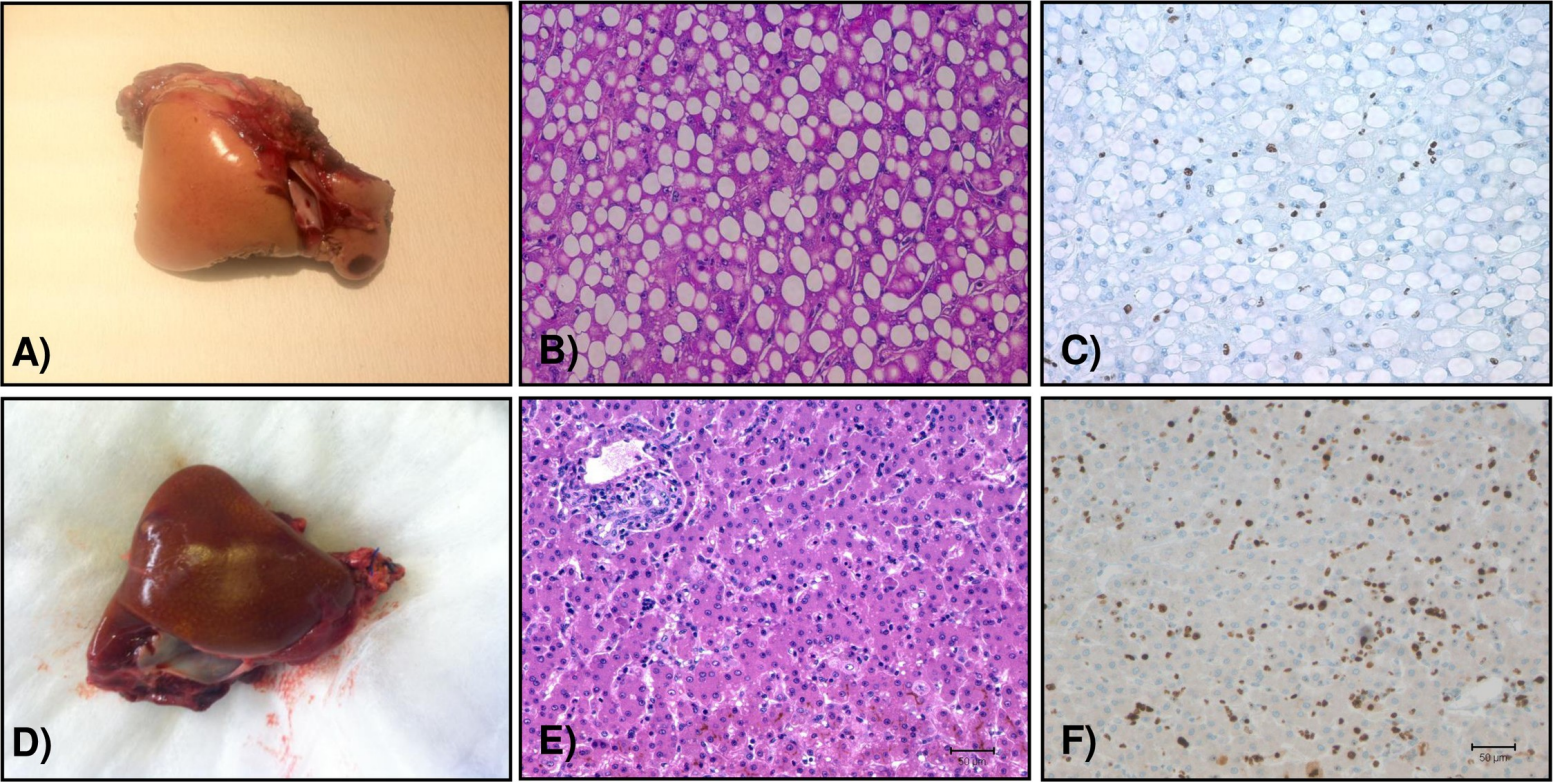
Macroscopic and microscopic analysis of remnant livers. (A) macroscopic image of one representative animal from the 90%Hx alone group at the time of autopsy with yellow appearance indicating steatosis, which was confirmed histologically (B). (C) Ki67 staining depicting very few replicating hepatocytes positive. Representative macroscopic (D), and histological images of the livers of animals undergoing 90%Hx +XHALT (E,F). Note the normal appearance of the remnant liver, no presence of steatosis and good amount of Ki67+ cells. Bars = 100μm.

The caudate lobe remnant in control animals was consistently yellow and friable at necropsy on POD3-5 (Fig 5A). Severe micro and macrosteatosis was confirmed by histology, and immunohistochemistry revealed very few Ki-67 positive cells (Fig 5B and 5C). In contrast, the caudate lobe remnants in animals that also received XHALT appeared grossly normal at necropsy on POD4, and on exploratory laparotomy POD6 and necropsy POD11 (Figs 5D, 3A and 3D and S1). Although these time points are later than necropsies performed on control animals, the two XHALT animals also underwent exploratory laparatomy on POD3 and 6, during the same time period as control animal underwent necropsy, and the native liver remnants appeared normal in color and texture at those earlier times, as well (Fig 3A). Biopsies of the caudate remnants taken at ex-lap and at necropsy on the XHALT animals showed normal parenchyma with no fat deposition and significant Ki-67 staining (Figs 3A and 5D–5F,).

### Xenogeneic liver graft outcomes

**Fig 6:**
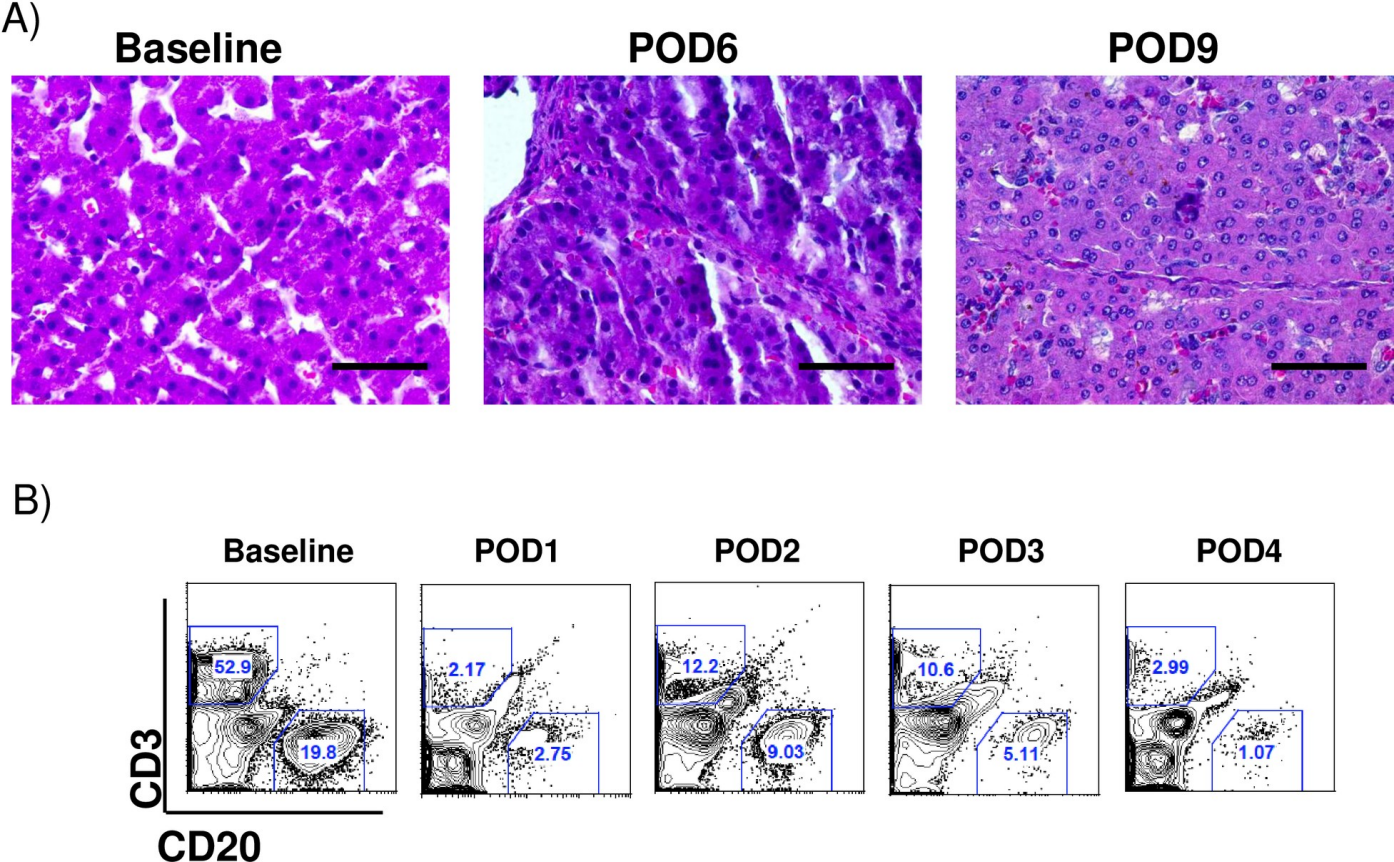
Rejection monitoring in xenogeneic auxiliary liver recipients. (A) Histological appearance of xenogeneic auxiliary liver from animals undergoing 90%Hx+ XHALT at the time of exploratory laparotomy and at graftectomy indicating absence of rejection signs. (B) representative Flow Cytometry data from peripheral blood CD3 and CD20 in from animals undergoing 90%Hx+ XHALT at different time points.

Early biopsies of xenogeneic livers showed mild thrombotic microangiopathy (TMA) that progressed to centrilobular necrosis in later samples (Fig 4). The grafts showed no signs of rejection on histology, which together with the low levels of circulating CD3 and CD20 cells (Fig 6), indicates that graft dysfunction was more due to TMA rather than rejection itself. Interestingly, the xenografts also showed a high percentage of Ki-67 cells (Fig 4B and 4C) and serum IL-6, a crucial cytokine initiating hepatocyte replication, was lower in XHALT animals than control animals (Fig 4A)

## Discussion

We found that a xenogeneic liver transplant from GTKO miniature swine was associated with prolonged survival and improve liver function parameters in baboons that had undergone 90% Hx. While it is not so surprising that an XHALT can support an NHP with only 10% of its liver, since orthotopic liver transplantation from pigs to baboons has resulted in survivals of over a week [10,15], it is remarkable that the presence of the XHALT prevented hepatocyte injury and steatosis and promoted hepatocyte replication in the native liver remnant. This is the first demonstration that providing liver support can mitigate injury and promote regeneration of a native small for size liver segment that would otherwise be insufficient to support life. XHALT recipients had lower serum ALT than 90% Hx only animals, and the native liver remnants had no histologic evidence of steatosis or inflammation (Figs 3 and S1), while 90% Hx only animals had severe micro and macrosteatosis and inflammation in their native liver remnants. Furthermore, XHALT recipients’ native liver remnants were regenerating at time points when control animals’ native liver remnants had no Ki-67 (POD 3–6) and continued to later time points (POD 4–11), when the control animals had already died. This suggests that XHALT has the potential to act as temporary *in vivo* liver support [16] and not only serve as a bridge to transplant, but also allow small segments to grow to adequate size or to allow native liver recovery from acute liver failure.

One serious limitation of this pilot study is the fact that the control group did not receive immunosuppression and neither undergo splenectomy with splenorenal shunt, in order to mimic the immunosuppressed state and portal decompression accompanying the XHALT in the animals that received that intervention. Therefore, it is difficult to distinguish the salutary effects of the xenoliver itself from potential benefits arising from suppression of inflammation and diversion of portal flow. Having observed clinical and histologic differences between the two groups in this study, it would be important to go back and perform additional controls to be able to clearly separate the effects of the three components comprising XHALT—the auxiliary xenohepatocyte mass, portal diversion, and immunosuppression-–as well as refine the XHALT procedure to obtain better xenograft function and survival. As we gain new knowledge and develop new technology for modifying xenolivers, this is becoming increasingly feasible.

There have been previous attempts to use xenogenic heterotopic liver transplantation as a bridge in humans, one with a chimpanzee liver and another with a pig liver [17,18]. Although both patients showed improvement in liver function and some clinical response, the first graft survived only 24 hours and the second patient died 34 hours after the procedure, so it was not possible to evaluate the effect of either on native liver regeneration or to be sure that the xenograft had improved the outcome. 11 day survival of the auxiliary liver xenograft, as in this study, is likely still not long enough for clinical application, as human livers generally take 1–2 months to grow back to full size after partial hepatectomy [19,20]. However, it was associated with prolonged survival and native hepatocyte replication. Furthermore, we have found that administration of exogenous human coagulation factors can prolong liver xenograft survival to 25 days, which probably is sufficient [10,11]. Our 10% native liver remnant was not able to achieve the effect of exogenous administration of coagulation factors, and the addition of exogenous coagulation factors to our small for size model could further improve outcomes in an XHALT model. Genetic modification aimed at controlling thrombotic microangiopathy and xenoimmune responses will likely have beneficial impact, as well.

Once the native liver grows to adequate size, the pig liver could subsequently be removed and immunosuppression withdrawn, obviating the adverse effects of long-term immunosuppression. In the case of supporting a small for size transplanted segment, immunosuppression could be decreased to standard levels for allografts, rather than the heavy immunosuppression currently required to maintain a xenograft.

It has been demonstrated that lipid accumulation following partial hepatectomy is specifically regulated and may be essential for normal liver regeneration [21]. However, dysregulation of this process results in lipid overloading and impaired hepatocyte DNA replication, potentially caused by ER stress [22]. We observed that animals undergoing 90% Hx showed severe steatosis and minimal hepatocyte replication (Fig 5A). With XHALT, the native liver remnant appeared normal macroscopically and histologically and hepatocyte replication was seen at both early and later time points (Figs 3 and 5 and S1).

Successful liver regeneration is a complex process involving many cytokines and growth factors from multiple cell types, acting in a delicate balance. After partial hepatectomy, the remnant liver undergoes changes in portal perfusion, which is thought to act as the initial stimuli to active innate immune cells such as Kupffer cells to secrete cytokines (IL-6 and TNF) that in turn will stimulate the hepatocytes, normally in G0, to enter G1 [23,24]. Following 90% Hx, with or without the addition of XHALT, there was an increase in IL-6 levels in all animals at just 3hrs following Hx, which is crucial in priming the hepatocytes for cell-cycle entry. Interestingly, in hepatectomy alone animals, IL-6 levels remain high, while they decreased over time in XHALT animals (Fig 4A). Interestingly, excess IL-6 can paradoxically block cell cycle entry and instead promote apoptosis [25], so it is possible that the continued elevation of IL-6 inhibits hepatocyte replication in the 90% Hx alone animals. It is not clear in our model whether the immunosuppressive regimen decreased IL-6 secretion, the xenogeneic liver promotes native liver regeneration by acting as an IL-6 sink, or if the presence of the xenograft limits the inflammatory response to liver injury and decreases production of IL-6.

It has also been shown that inflammatory mediators such as IL-6 are increased in serum from patients with NASH and these patients are also known to have disturbed liver regeneration [26,27]. In the presence of fatty hepatocytes, intracellular fatty acids foster the production of reactive oxygen species (ROS) augmenting mitochondrial oxidative stress and lipid peroxidation [28,29]. Moreover, steatotic livers have impaired antioxidant scavenging ability of free radicals triggering ROS buildup, leading to the activation of innate immune cells such as Kupffer cells (KC), which produce pro-inflammatory cytokines such as tumor necrosis factor (TNF), IL-6 and IL-1, further exacerbating hepatocellular damage in steatotic livers [28,29]. Animals undergoing 90% hepatectomy alone, presented with significantly higher degree of steatosis compared to animals undergoing XHALT (Fig 5A and 5B). The severe inflammatory reaction may exacerbate the decreased functional capacity of the steatotic remnant. In XHALT, lower levels of IL-6 might also limit the systemic inflammatory response, contributing to prolonged survival.

In summary, XHALT from Gal-KO pigs to baboons with otherwise lethal PHLF has the potential to act through multiple pathways to result in our preliminary finding that it is associated with prolonged survival, decreased native hepatocyte injury, and increased native hepatocyte replication. Further work needs to be done to determine whether temporary auxiliary liver xenografts could support patients with inadequate liver mass, whether from acute liver injury, hepatectomy, or small segment transplantation, through native liver recovery. New technologies and reports from other groups have made the prospect of clinical xenotransplantation a more realistic option. There are 3 major hurdles to overcome before we can use a porcine liver clinically: 1) immune response 2) physiological incompatibilities between the porcine organ and the human recipient and 3) the risk of transmitting zoonotic pathogens from pig to humans. With the advances in gene editing tools, it is now much more feasible to produce knock out or knock in pigs which could alleviate the physiological and immunological hurdles [30]. This technology has already been used to eliminate PERV expression [31]. In addition, the use of new immunosuppressive agents such as costimulatory blockade, have enabled prolonged survival of extra-hepatic xenografts for several months—long enough to serve as temporary support to bridge the patient until a destination organ becomes available [10,32,33]

## Supporting information

S1 Fig Native liver evaluation. Histological appearance of the native liver from animal B347 undergoing 90%Hx+ XHALT at baseline, the time of exploratory laparotomy (POD6) and on autopsy (POD11). Both time points demonstrating normal healthy-looking livers with no evidence of steatosis or inflammation (40x magnification).
(TIF)

## Abbreviations

90%Hx: 90% hepatectomy
XHALT: Xenogeneic Heterotopic Auxiliary Liver Transplantation
PHLF: Post-Hepatectomy Liver Failure
SFSS: small-for-size-syndrome
LDLT: living donor liver transplantation
GT-KO: a-1,3-galactosyltransferase gene-knockout
CVF: Cobra Venom Factor
ATG: Anti-thymocyte globulin
LFTs: Liver Failure Tests
FFP: Fresh Frozen Plasma
pRBCs: Packed Red Blood Cells
UW: University of Wisconsin solution
PV: portal vein
SV: the splenic vein
SMV: superior mesenteric veins

## References

[1] Golriz M, Majlesara A, El Sakka S, Ashrafi M, Arwin J, Fard N, et al Small for Size and Flow (SFSF) syndrome: An alternative description for posthepatectomy liver failure. Clin Res Hepatol Gastroenterol. 2016 6 3;40(3):267–75. 10.1016/j.clinre.2015.06.024 26516057 10.1016/j.clinre.2015.06.024

[2] Lo C, Fan S, Liu C, Chan J, Lam B, Lau G, et al Minimum graft size for successful living donor liver transplantation. Transplantation. 1999;68(8):1112–6. 10551638 10.1097/00007890-199910270-00009

[3] Ben‐Haim M, Emre S, Fishbein T, Sheiner P, Bodian C, Kim‐Schluger L, et al Critical graft size in adult‐to‐adult living donor liver transplantation: Impact of the recipient’s disease. Liver Transplantation. 2001;7(11):948–53. 10.1053/jlts.2001.29033 11699030 10.1053/jlts.2001.29033

[4] Marubashi S, Nagano H, Eguchi H, Wada H, Asaoka T, Tomimaru Y, et al Minimum graft size calculated from preoperative recipient status in living donor liver transplantation. Liver Transpl. 2016 5;22(5):599–606. 10.1002/lt.24388 26684397 10.1002/lt.24388

[5] Kiuchi T, Tanaka K, Ito T, Oike F, Ogura Y, Fujimoto Y, et al Small‐for‐size graft in living donor liver transplantation: How far should we go? Liver Transplantation. 2003;9(9):S29–S35. 10.1053/jlts.2003.50198 12942476 10.1053/jlts.2003.50198

[6] Hashikura Y, Ichida T, Umeshita K, Kawasaki S, Mizokami M, Mochida S, et al Donor Complications Associated With Living Donor Liver Transplantation in Japan. Transplantation. 2009;88(1):110 10.1097/TP.0b013e3181aaccb0 19584689 10.1097/TP.0b013e3181aaccb0

[7] Akabayashi A, Slingsby B, Fujita M. The first donor death after living-related liver transplantation in Japan. Transplantation. 2004;77(4):634. 10.1097/01.tp.0000115342.98226.7c 15084953

[8] Ekser B, Gridelli B, Veroux M, Cooper DK. Clinical pig liver xenotransplantation: how far do we have to go? Xenotransplantation. 2011 1 6;18(3):158–67. 10.1111/j.1399-3089.2011.00642.x 21696445 10.1111/j.1399-3089.2011.00642.x

[9] Kolber-Simonds D, Lai L, Watt SR, Denaro M, Arn S, Augenstein ML, et al Production of alpha-1,3-galactosyltransferase null pigs by means of nuclear transfer with fibroblasts bearing loss of heterozygosity mutations. Proc Natl Acad Sci USA. 2004 5 2;101(19):7335–40. 10.1073/pnas.0307819101 15123792 10.1073/pnas.0307819101 PMC409919

[10] Shah JA, Navarro-Alvarez N, DeFazio M, Rosales IA, Elias N, Yeh H, et al A Bridge to Somewhere: 25-day Survival After Pig-to-Baboon Liver Xenotransplantation. Ann Surg. 2016 6 3;263(6):1069–71. 10.1097/SLA.0000000000001659 26825261 10.1097/SLA.0000000000001659

[11] Shah JA, Patel MS, Elias N, Navarro-Alvarez N, Rosales I, Wilkinson RA, et al Prolonged Survival Following Pig-to-Primate Liver Xenotransplantation Utilizing Exogenous Coagulation Factors and Costimulation Blockade. Am J Transplant. 2017 8 2;17(8):2178–85. 10.1111/ajt.14341 28489305 10.1111/ajt.14341 PMC5519420

[12] Kim K, Schuetz C, Elias N, Veillette GR, Wamala I, Varma M, et al Up to 9-day survival and control of thrombocytopenia following alpha1,3-galactosyl transferase knockout swine liver xenotransplantation in baboons. Xenotransplantation. 2012 1;19(4):256–64. 10.1111/j.1399-3089.2012.00717.x 22909139 10.1111/j.1399-3089.2012.00717.x PMC3655405

[13] Machaidze Z, Yeh H, Wei L, Schuetz C, Carvello M, Sgroi A, et al Testing of microencapsulated porcine hepatocytes in a new model of fulminant liver failure in baboons. Xenotransplantation. 2017 5 1;24(3). 10.1111/xen.12297 28261903

[14] Yeh H, Machaidze Z, Wamala I, Fraser JW, Navarro-Alvarez N, Kim K, et al Increased transfusion-free survival following auxiliary pig liver xenotransplantation. Xenotransplantation. 2014 1 3;21(5):454–64. 10.1111/xen.12111 25130043 10.1111/xen.12111

[15] Navarro-Alvarez N, Shah JA, Zhu A, Ligocka J, Yeh H, Elias N, et al The Effects of Exogenous Administration of Human Coagulation Factors Following Pig-to-Baboon Liver Xenotransplantation. Am J Transplant. 2016 6 3;16(6):1715–25. 10.1111/ajt.13647 26613235 10.1111/ajt.13647 PMC4874924

[16] Ekser B, Gridelli B, Tector J, Cooper D. Pig Liver Xenotransplantation as a Bridge to Allotransplantation: Which Patients Might Benefit? Transplantation. 2009;88(9):1041 10.1097/TP.0b013e3181ba0555 19898198 10.1097/TP.0b013e3181ba0555 PMC2778799

[17] Hara H, Gridelli B, Lin Y, Marcos A, Cooper D. Liver xenografts for the treatment of acute liver failure: Clinical and experimental experience and remaining immunologic barriers. Liver Transplantation. 2008;14(4):425–34. 10.1002/lt.21476 18383106 10.1002/lt.21476

[18] Makowa, Cramer, Hoffman, Breda, Sher, Eiras-Hreha, et al The use of a pig liver xenograft for temporary support of a patient with fulminant hepatic failure. Transplantation. 1995;59(12):1654–9. 7604434 10.1097/00007890-199506270-00002

[19] Cheng Y-F, Huang T-L, Chen T-Y, Tsang, Ou H-Y, Yu C-Y, et al Liver graft regeneration in right lobe adult living donor liver transplantation. American journal of transplantation: official journal of the American Society of Transplantation and the American Society of Transplant Surgeons. 2009;9(6):1382–8. 10.1111/j.1600-6143.2009.02626.x 19459827

[20] Olthoff K. Hepatic regeneration in living donor liver transplantation. Liver transplantation: official publication of the American Association for the Study of Liver Diseases and the International Liver Transplantation Society. 2003;9(10 Suppl 2):S35–41. 10.1053/jlts.2003.50229 14528426

[21] Shteyer E, Liao Y, Muglia L, Hruz P, Rudnick D. Disruption of hepatic adipogenesis is associated with impaired liver regeneration in mice. Hepatology. 2004;40(6):1322–32. 10.1002/hep.20462 15565660 10.1002/hep.20462

[22] Hamano M, Ezaki H, Kiso S, Furuta K, Egawa M, Kizu T, et al Lipid overloading during liver regeneration causes delayed hepatocyte DNA replication by increasing ER stress in mice with simple hepatic steatosis. Journal of Gastroenterology. 2014;49(2):305–16. 10.1007/s00535-013-0780-7 23512345 10.1007/s00535-013-0780-7 PMC3925298

[23] Michalopoulos, DeFrances. Liver regeneration. Science (New York, NY). 1997;276(5309):60–6. 10.1126/science.276.5309.60 9082986

[24] Michalopoulos G. Advances in liver regeneration. Expert review of gastroenterology & hepatology. 2014;8(8):897–907. 24964729 10.1586/17474124.2014.934358

[25] Wüstefeld, Rakemann, Kubicka, Manns, Trautwein. Hyperstimulation with interleukin 6 inhibits cell cycle progression after hepatectomy in mice. Hepatology (Baltimore, Md). 2000;32(3):514–22. 10.1053/jhep.2000.16604 10960443

[26] Coulon S, Francque S, Colle I, Verrijken A, Blomme B, Heindryckx F, et al Evaluation of inflammatory and angiogenic factors in patients with non-alcoholic fatty liver disease. Cytokine. 2012;59(2):442–9. 10.1016/j.cyto.2012.05.001 22658783 10.1016/j.cyto.2012.05.001

[27] Abiru S, Migita K, Maeda Y, Daikoku M, Ito M, Ohata K, et al Serum cytokine and soluble cytokine receptor levels in patients with non‐alcoholic steatohepatitis. Liver International. 2006;26(1):39–45. 10.1111/j.1478-3231.2005.01191.x 16420507 10.1111/j.1478-3231.2005.01191.x

[28] Sumida Y, Niki E, Naito Y, Yoshikawa T. Involvement of free radicals and oxidative stress in NAFLD/NASH. Free radical research. 2013 12 4;47(11):869–80. 10.3109/10715762.2013.837577 24004441 10.3109/10715762.2013.837577

[29] Gawrieh S, Opara E, Koch T. Oxidative stress in nonalcoholic fatty liver disease: pathogenesis and antioxidant therapies. Journal of investigative medicine: the official publication of the American Federation for Clinical Research. 2004;52(8):506–14. 15682682 10.1136/jim-52-08-22

[30] Butler JR, Ladowski JM, Martens GR, Tector M, Tector AJ. Recent advances in genome editing and creation of genetically modified pigs. Int J Surg. 2015 11;23(Pt B):217–22. 10.1016/j.ijsu.2015.07.684 26231992 10.1016/j.ijsu.2015.07.684

[31] Yang L, Güell M, Niu D, George H, Lesha E, Grishin D, et al Genome-wide inactivation of porcine endogenous retroviruses (PERVs). Science. 2015 11 5;350(6264):1101–4. 10.1126/science.aad1191 26456528 10.1126/science.aad1191

[32] Ekser B, Markmann J, Tector J. Current status of pig liver xenotransplantation. International Journal of Surgery. 2015;23(Pt B):240–6. 10.1016/j.ijsu.2015.06.083 26190837 10.1016/j.ijsu.2015.06.083

[33] Iwase H, Liu H, Wijkstrom M, Zhou H, Singh J, Hara H, et al Pig kidney graft survival in a baboon for 136 days: longest life-supporting organ graft survival to date. Xenotransplantation. 2015 1 4;22(4):302–9. 10.1111/xen.12174 26130164 10.1111/xen.12174 PMC4519393

