# Supplementary figures and images for: Xenogeneic Heterotopic Auxiliary Liver transplantation (XHALT) promotes native liver regeneration in a Post-Hepatectomy Liver failure model
Source: PLoS One. 2018 Nov 21;13(11):e0207272. doi: 10.1371/journal.pone.0207272 (PMC6248961; doi:10.1371/journal.pone.0207272)

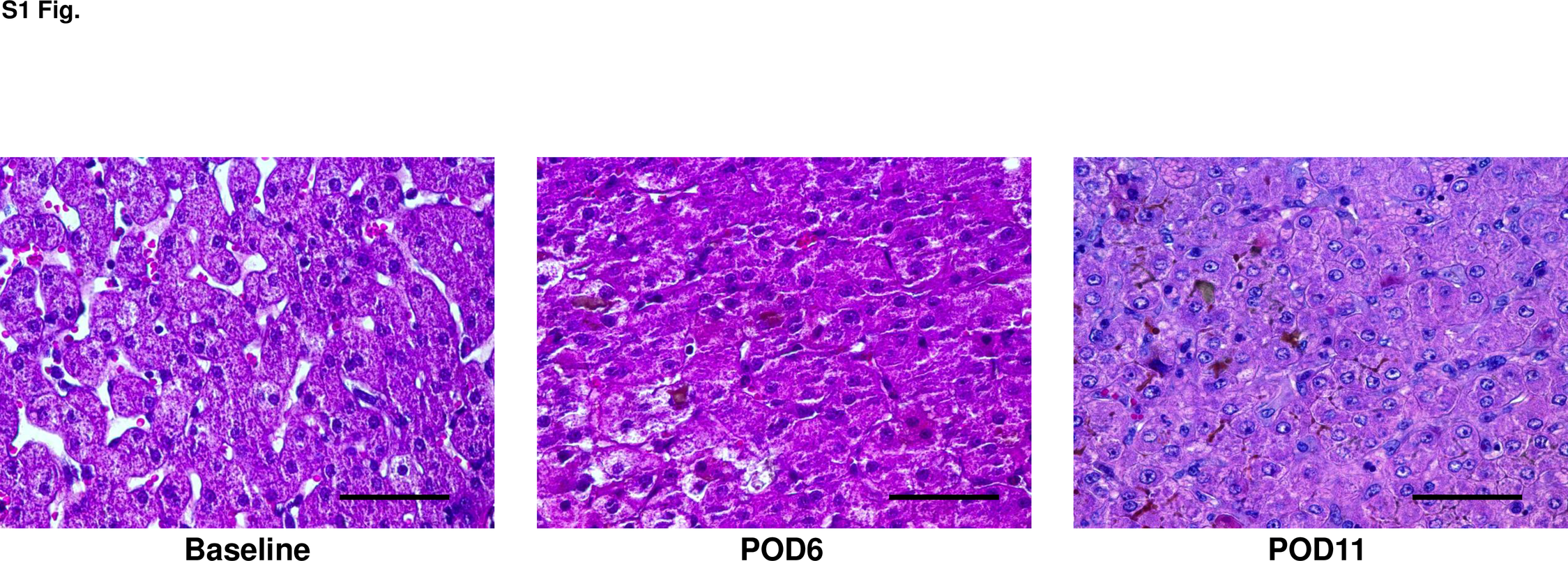

Supplement: S1 Fig — Histological appearance of the native liver from animal B347 undergoing 90%Hx+ XHALT at baseline, the time of exploratory laparotomy (POD6) and on autopsy (POD11). Both time points demonstrating normal healthy-looking livers with no evidence of steatosis or inflammation (40x magnification). (TIF) [file pone.0207272.s001.tif]
